# Supplementary material for: Global warming-induced Asian hydrological climate transition across the Miocene–Pliocene boundary
Source: Nat Commun. 2021 Nov 26;12:6935. doi: 10.1038/s41467-021-27054-5 (PMC8626456; doi:10.1038/s41467-021-27054-5)
Supplement: Supplementary file 1 — Supplementary Information [file 41467_2021_27054_MOESM1_ESM.pdf]

Supplementary Information

Global warming-induced Asian hydrological climate transition across the Miocene–Pliocene boundary

Ao et al.

Supplementary Note 1: Justification of proxies used for summer monsoon precipitation

Aeolian deposits on the Chinese Loess Plateau (CLP), including Quaternary loess and late Oligocene–Pliocene red clay, reflect dust transportation from higher-latitude Central Asian arid regions primarily by northwesterly winter monsoon winds, as indicated by a northwest-southeast grain size decrease across the CLP[1-4]. Soil development on the aeolian substrate is related to southeasterly moisture transportation and precipitation associated with inland summer monsoon penetration[1-5]. These aeolian deposits provide an outstanding terrestrial archive of past climate changes linked to both high- and low-latitude processes, including tracking of both summer and winter monsoon variations at the same time.

Al/Na, Rb/Sr, and lightness of both Quaternary loess and underlying Neogene red clay on the CLP are used routinely as proxies of regional summer monsoon precipitation[4,6-10]. Higher precipitation results in stronger pedogenic weathering, depletes mobile Na and Sr, and enriches insoluble Al and Rb, leading to higher Al/Na and Rb/Sr values[4,7,9]. Higher precipitation also causes pedogenic enrichment of dark-coloured minerals (fine-grained magnetite, clay minerals, and organic matter) and (light coloured) carbonate leaching, which results in more saturated red soil colouration, with lower lightness[6,8]. For the Quaternary loess sequence, intercalated interglacial palaeosols have higher Al/Na and Rb/Sr values and lower lightness values than glacial loess layers, and are associated with stronger pedogenesis and higher precipitation[7-9] (Supplementary Fig. 2).

Like the Quaternary loess, the underlying Neogene red clay reflects similar dust transportation primarily by the northwesterly winter monsoon from arid regions to the west and north[1-4]. Warmer and wetter Neogene conditions resulted in much stronger pedogenesis and more intense colouration than in the overlying Quaternary loess-palaeosol sequence (Fig. 1b). Unlike the Quaternary loess that is characterized by orbital alternations between yellow loess and light red palaeosols, the underlying red clay is dominated by orbitally driven alternations between brownish palaeosols and carbonate-nodule-rich light red palaeosols in the late Miocene, and by increasingly darker red palaeosols in the Pliocene. Intercalated light red palaeosols, which formed during orbitally induced relatively cold periods within the late Miocene, are still redder than the light red Quaternary palaeosols that formed during warm interglacials (Fig. 1b; Supplementary Fig. 1). Yellow loess layers typical of the Quaternary are absent in the late Miocene–Pliocene Shilou red clay. This pattern is consistent with red clay formation under warmer and more sustained pre-Quaternary moist conditions.

As expected, brownish and darker red palaeosols, which formed under higher precipitation and stronger pedogenic weathering conditions, have higher Al/Na and Rb/Sr values and lower lightness values than the light red carbonate-nodule-rich palaeosols, which formed under lower precipitation and weaker pedogenic weathering conditions (Fig. 2a–d). CLP loess and red clay accumulation reflects recycling of dusts transported originally from Asian mountains to arid regions located to the north and west of the CLP[11], with Al/Na, Rb/Sr, and lightness variations reflecting primarily post-depositional pedogenesis, with only a slight pre-depositional pedogenic influence from dust sources[4,7-10]. In addition, large amplitude pedogenic changes in CLP aeolian sequences are unlikely to be controlled by dust accumulation rate changes. For example, CLP palaeosols S_1, S_2, S_3, and S_4 have different accumulation rates during different interglacials within the last 400 kyr, but they have almost identical pedogenic magnetic susceptibility enhancement that indicates similar monsoon precipitation amount[12]. Thus, Al/Na, Rb/Sr, and lightness of Neogene red clay and Quaternary loess-palaeosol sequences across the CLP are used routinely as proxies of regional summer monsoon precipitation, and their close relationship with precipitation is well documented in many previous studies[4,6-10].

Supplementary Note 2: Magnetostratigraphy

Xu et al.[13] reported the first palaeomagnetic record of the Shilou red clay section based on ~300 samples that were measured at >20 cm stratigraphic intervals using a JR-6A spinner magnetometer. The spinner magnetometer was not housed in a magnetically shielded space, which potentially results in relatively lower signal-to-noise ratios than our later measurements with a 3-axis 2-G Enterprises cryogenic magnetometer that is housed within a magnetically shielded space. Xu et al.[13] interpreted their magnetic polarity record to span

from polarity subchron C5n.2n to C2An.1n, with a ~11–2.6 Ma age range. However, they defined some polarity zones based on only one sample, which is usually deemed insufficient for robust polarity zone definition. This may have artificially increased the number of polarity zones in the section and caused the section to be interpreted to be older and to span a longer duration. Anwar et al.[14] interpreted the same palaeomagnetic record markedly differently to span from C3n.4n to C2An.1n, with an age of ~5.2–2.6 Ma. However, this reinterpretation has many unmatched magnetostratigraphic correlations. For example, they interpreted some intervals as excursions that are absent in the geomagnetic polarity time scale (GPTS), and correlated two polarity zones to a single polarity chron in the GPTS in several intervals. This caused the Shilou red clay to appear to be younger and span a shorter time interval. In addition, some of their interpreted excursions have relatively long durations, which is inconsistent with the short expected durations of palaeomagnetic excursions.

We recently carried out a more detailed palaeomagnetic investigation of 978 new samples from the Shilou red clay taken at 5–10 cm stratigraphic intervals and measured with a 3-axis 2-G Enterprises Model 755-R cryogenic magnetometer housed in a magnetically shielded space at the Institute of Earth Environment, Chinese Academy of Sciences, Xi'an, China[1]. The updated magnetostratigraphy has a more than three times higher sampling resolution and better signal-to-noise ratio, which significantly improves the polarity zone definition compared to previous studies[13,14], and enables unequivocal magnetostratigraphic correlation to the GPTS (C4r.1r–C2An.1n, ~8.2–2.6 Ma). This updated age model is further supported by our recent publication[15], which incorporates all comments on previous versions of the Shilou age model, and reaffirms that our updated magnetostratigraphy is a robust interpretation. Our updated magnetochronology[1] has the same polarity zone structure as the GPTS and is consistent with the palaeomagnetic polarity zones of the GPTS, and with the magnetic susceptibility, lithological, and mammal fossil stratigraphies of other red clay sections across the CLP, which have been discussed at length in our recent studies[1,5,15]. Thus, we use here the updated magnetochronology for the Shilou red clay section[1].

Supplementary Figures

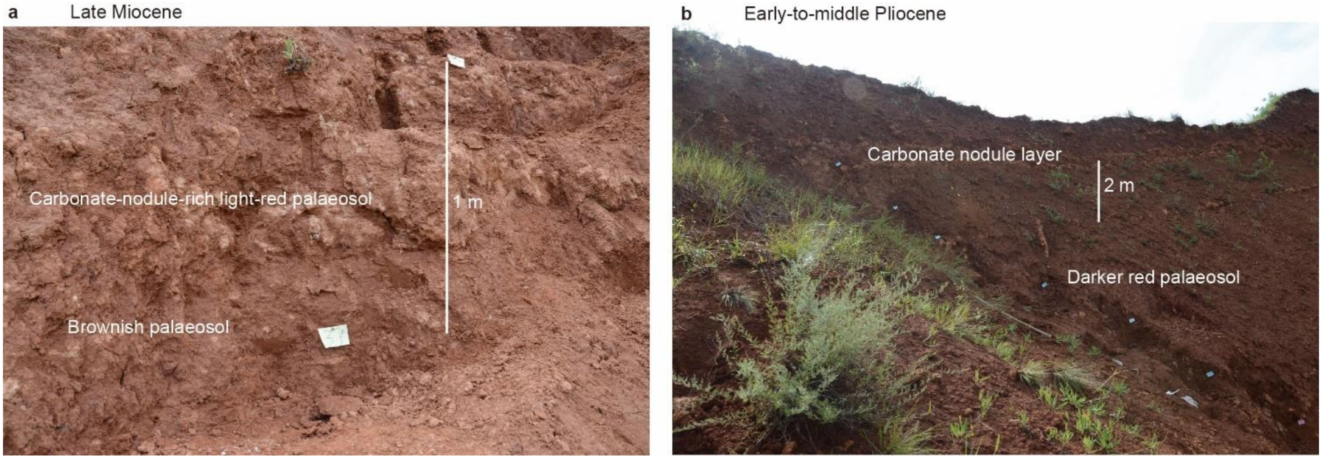

Supplementary Fig. 1. Field photographs of lithologies in the Shilou red clay section (taken by H. Ao). (**a**) Alternations between late Miocene brownish palaeosols and carbonate-nodule-rich light-red palaeosols. (**b**) Early-to-middle Pliocene darker red palaeosols with only a few thin (0.2–0.4 m) carbonate nodule layers.

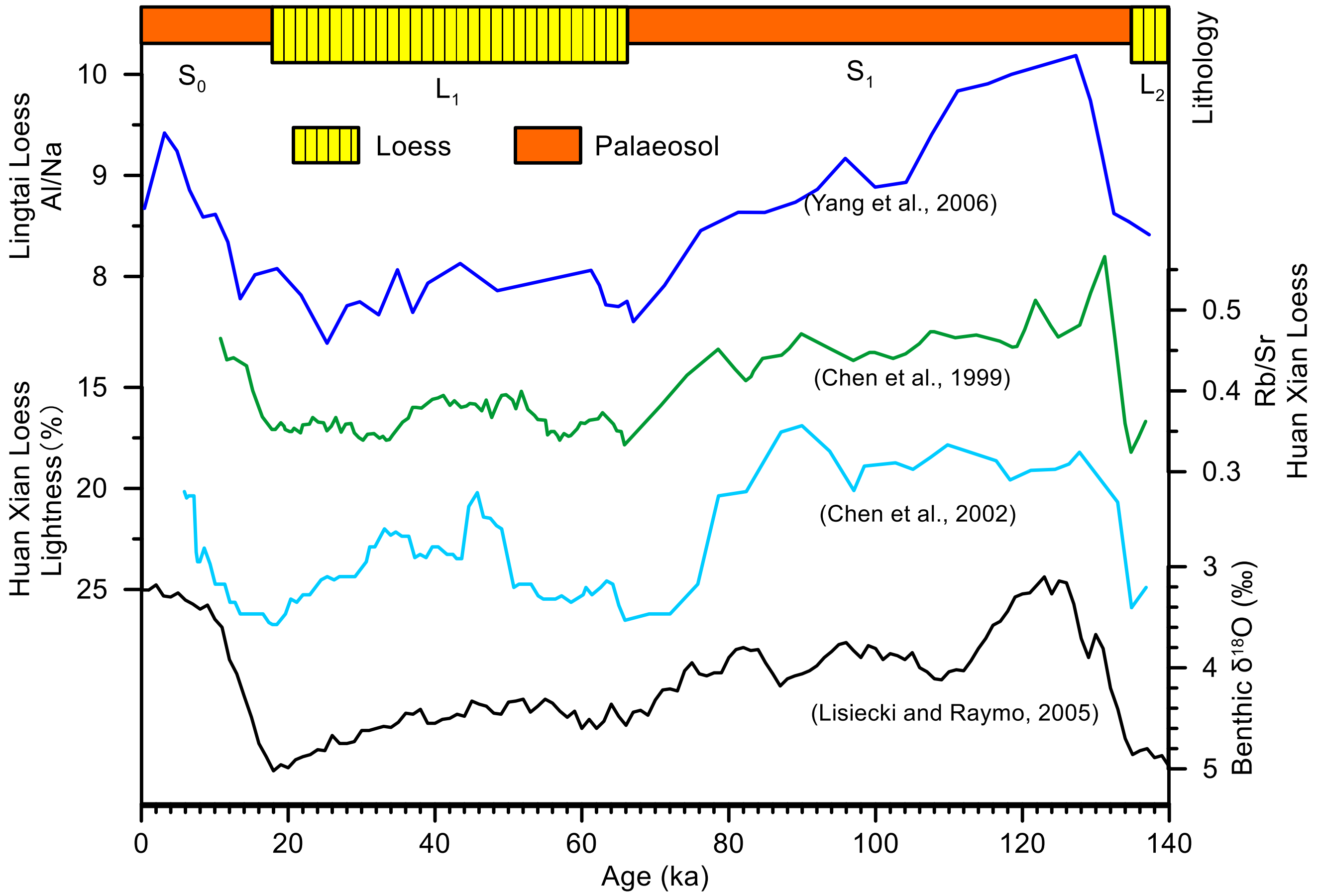

Supplementary Fig. 2. Comparison of late Quaternary loess proxy variability. Loess Al/Na, Rb/Sr, and lightness variability on the northern CLP[7-9] are compared with the marine benthic $\delta^{18}O$ stack[16] over the past 140 kyr. Interglacial palaeosols S_0 and S_1 have higher Al/Na and Rb/Sr values, and lower lightness values, and correspond to higher summer monsoon precipitation, relative to the intercalated glacial L_1 and L_2 loess units.

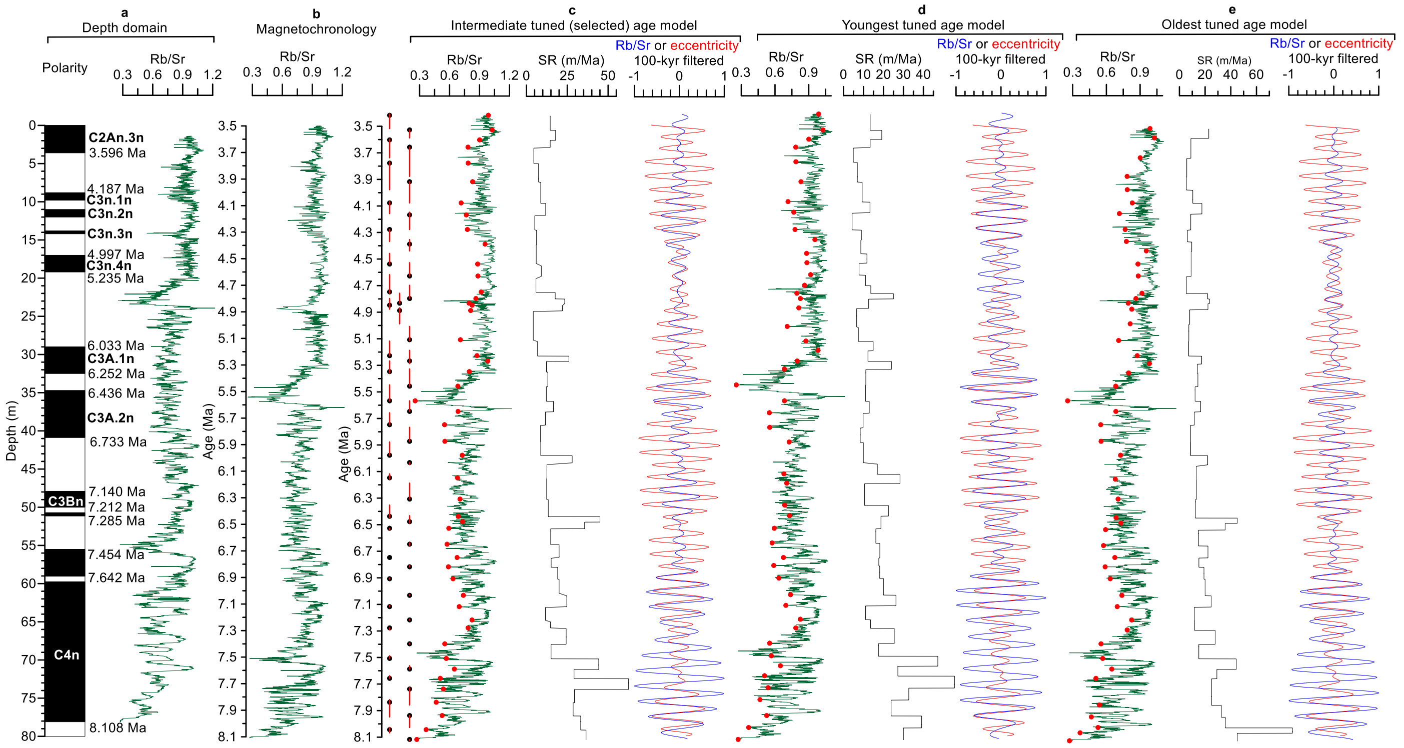

Supplementary Fig. 3. Construction of a refined age model for the Shilou red clay through orbital tuning. The astronomical chronology was generated by tuning 100-kyr components filtered from the Rb/Sr record to 100-kyr eccentricity[17]. (**a**) Palaeomagnetic polarity zones[1] and Rb/Sr record plotted against depth. Ages for magnetic reversal boundaries refer to the 2012 GPTS[18]. (**b**) Rb/Sr record plotted on an initial timescale based on the magnetochronology of Ao et al.[1] Tuning points with age uncertainties (black dots with red vertical bars, see methods for detailed definition of age uncertainties), Rb/Sr record (green curve) with tuning points (red dots), linear sedimentation rate (SR) between age control points, and comparison of the 100-kyr component filtered from the tuned Rb/Sr record (blue curve) and orbital eccentricity[17] (red curve) in the (**c**) intermediate (finally selected), (**d**) youngest, (**e**) oldest tuned age models.

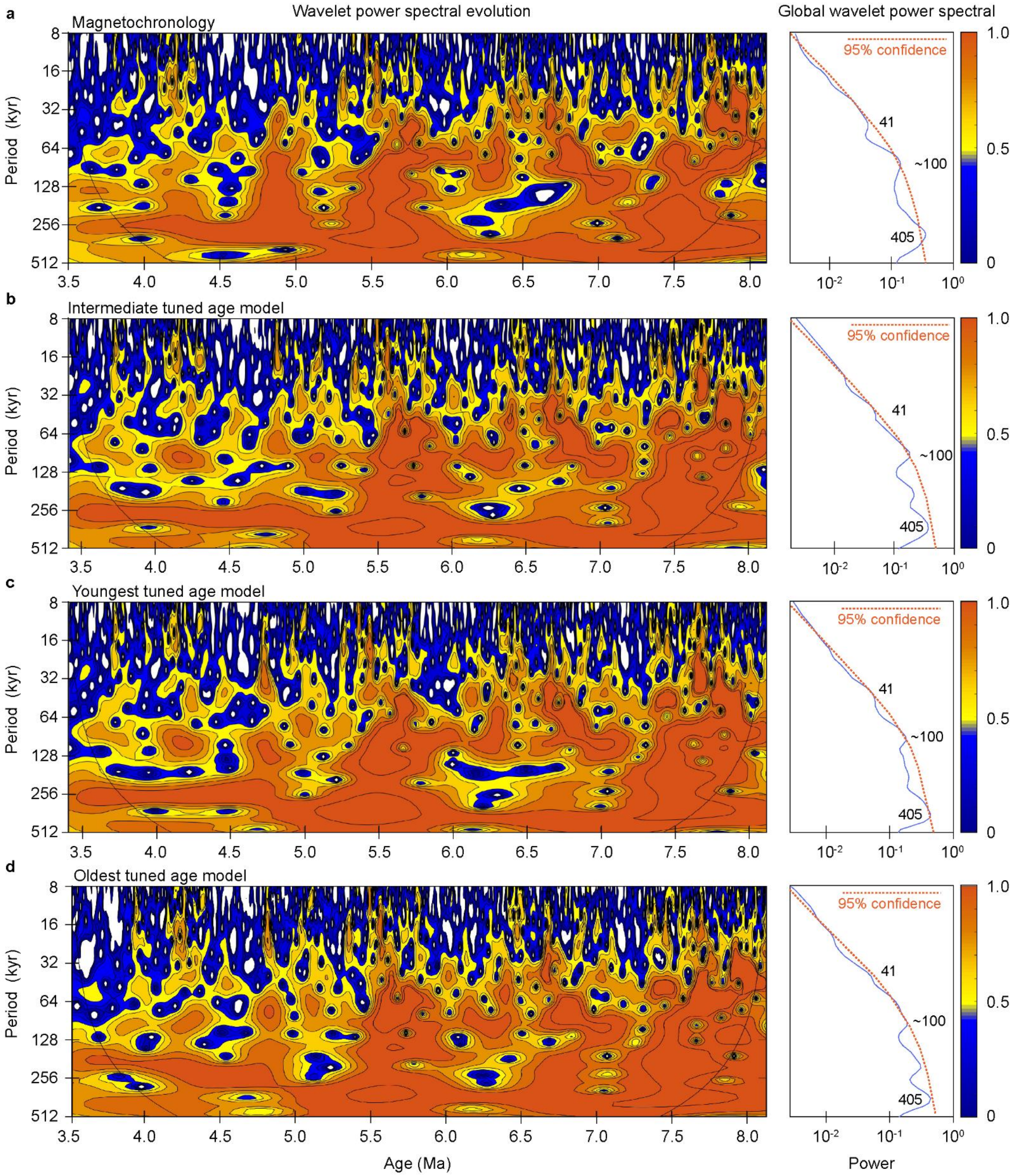

Supplementary Fig. 4. Orbital Rb/Sr variability in various age models. Wavelet power spectral evolution and global wavelet power spectra for the Shilou red clay Rb/Sr record for (**a**) the original magnetochronology, and (**b**) intermediate (finally selected), (**c**) youngest, and (**d**) oldest tuned age models.

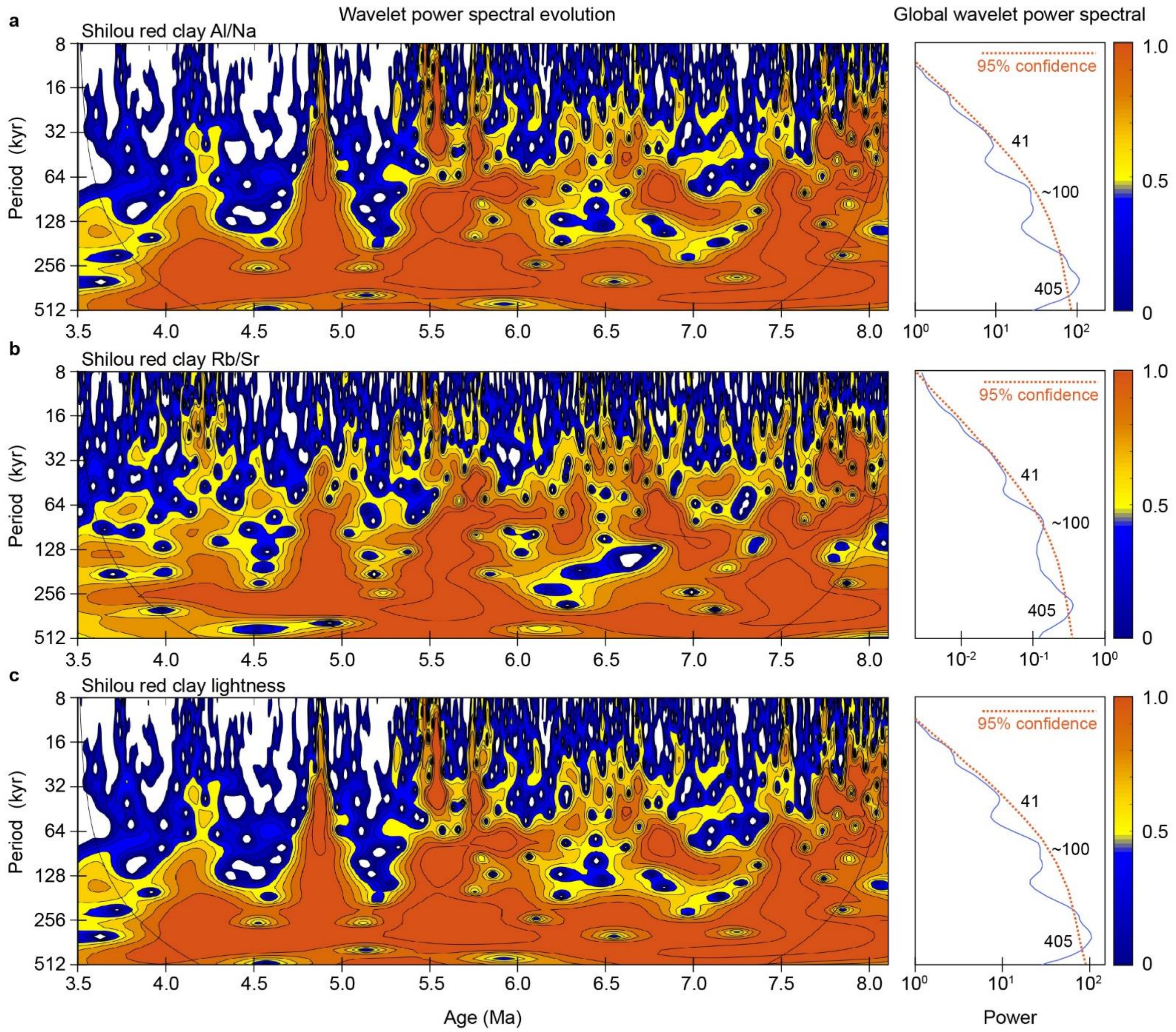

Supplementary Fig. 5. Orbital variability of Asian summer monsoon records from the Shilou red clay in the magnetochronology. Wavelet power spectral evolution and global wavelet power spectra for (**a**) Al/Na, (**b**) Rb/Sr, and (**c**) lightness records.

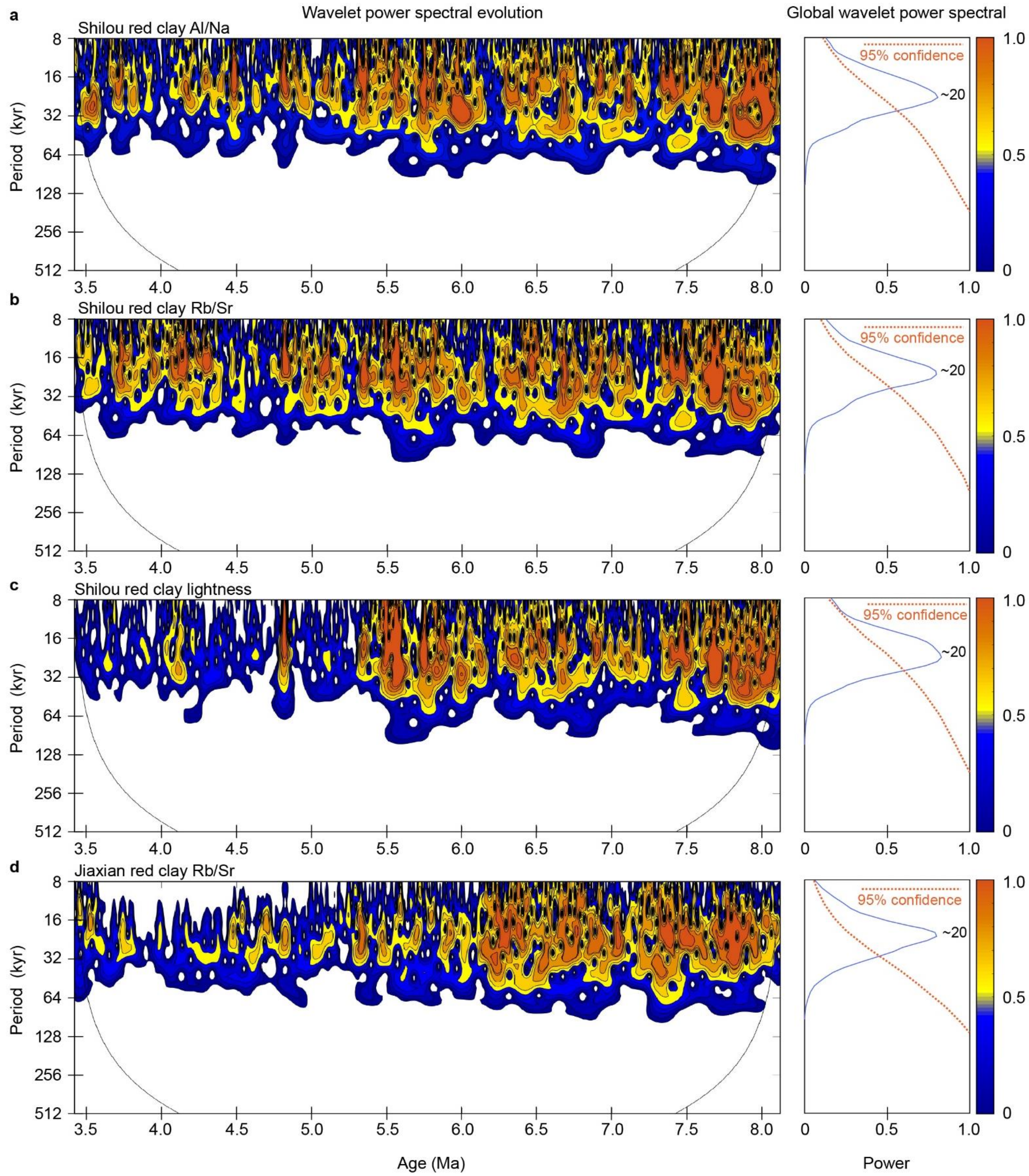

Supplementary Fig. 6. Precession variability in the red clay of the Chinese Loess Plateau. (**a**) Wavelet power spectral evolution and global wavelet power spectrum for (**a**) Al/Na, (**b**) Rb/Sr, and (**c**) lightness for the Shilou section, and (**d**) Rb/Sr for the Jiaxian section[10] after removal of longer (>40-kyr) periodicities (obliquity and eccentricity) from the data with local regression smoothing using the Acycle software[19] to assess a potential precession signal. Before removal, the precession signal is overprinted by stronger eccentricity and obliquity. After removal, a distinct precession signal appears.

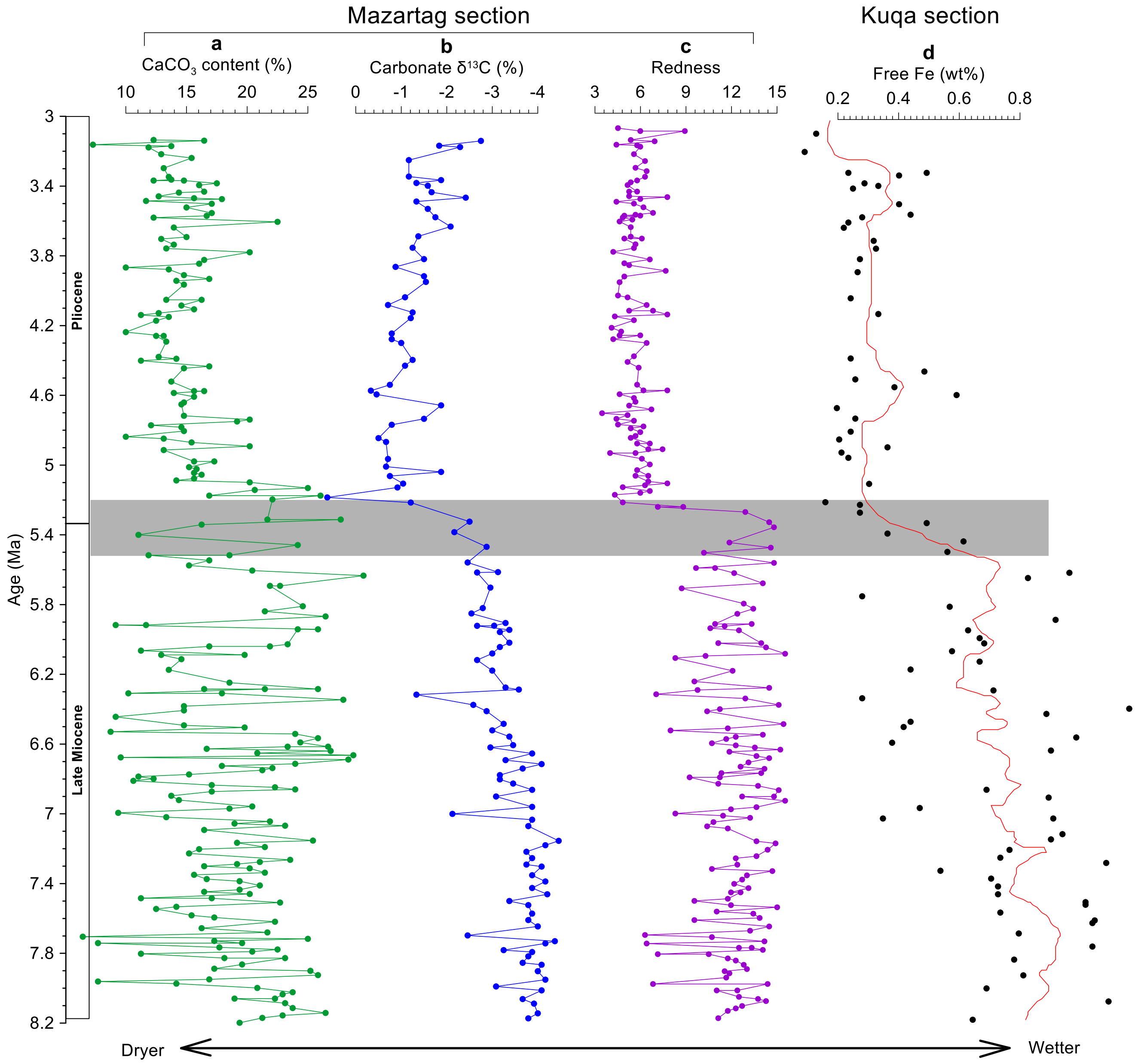

Supplementary Fig. 7. Late Miocene to Pliocene hydroclimate variability in arid Central Asia. (**a**) $CaCO_3$ content, (**b**) carbonate $\delta^{13}C$, (**c**) redness, and (**d**) free Fe concentration (red line is a smoothing trend) records from the Tarim Basin[20]. Decreases in $CaCO_3$ content, redness, and free Fe concentration, and a positive carbonate $\delta^{13}C$ shift across the MPB (shaded) are consistent with a marked sedimentological transition from predominantly lacustrine to fluvial-aeolian conditions, which correspond to a shift to drier conditions.

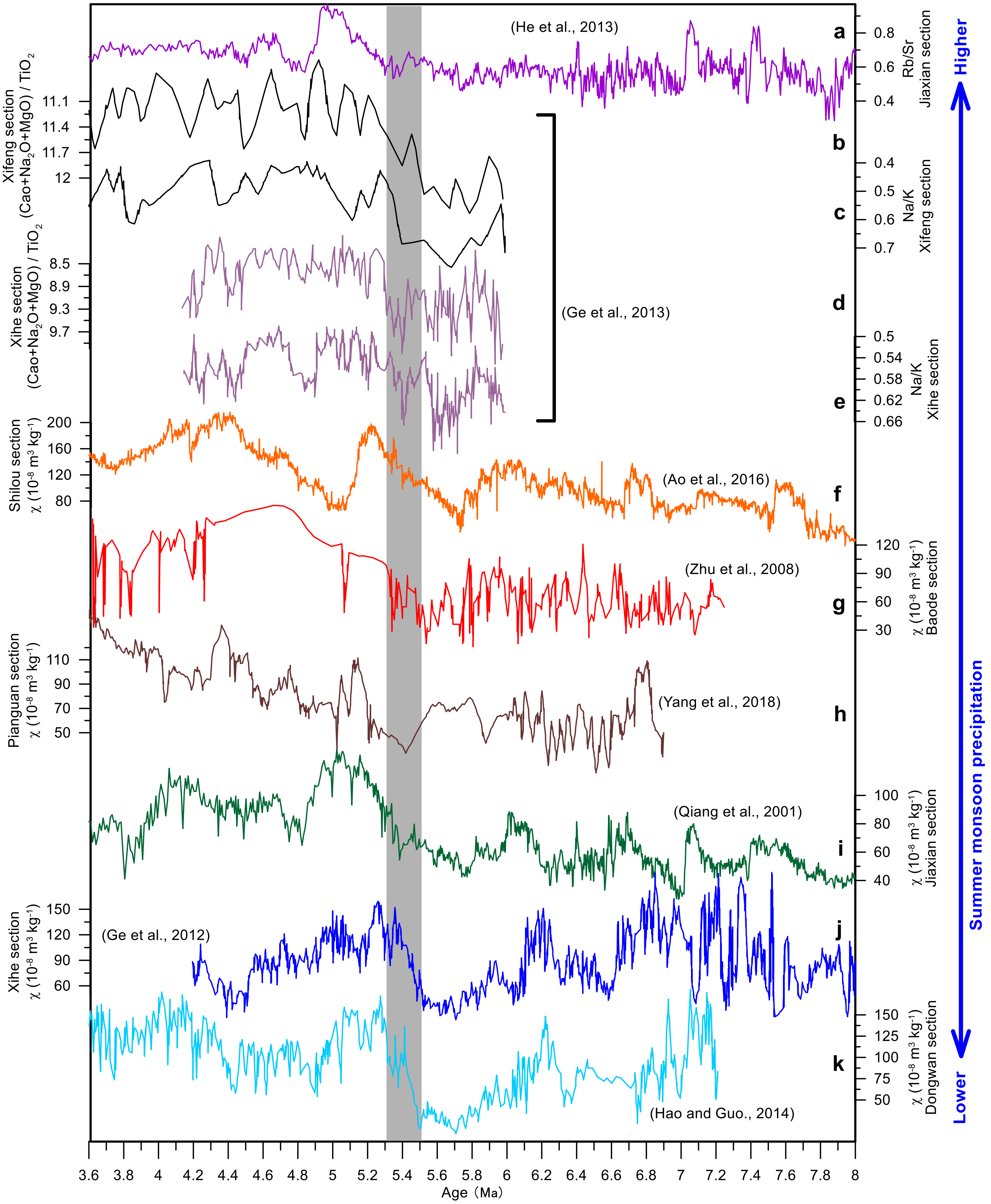

Supplementary Fig. 8. Chinese Loess Plateau red clay records of summer monsoon variability. **(a)** Jiaxian red clay Rb/Sr record[10]. **(b–e)** $(CaO + Na_2O + MgO)/TiO_2$ and Na/K records from the Xifeng and Xihe red clay sections[21]. Red clay magnetic susceptibility (χ) records from the **(f)** Shilou[1], **(g)** Baode[22], **(h)** Pianguan[23], **(i)** Jiaxian[24], **(j)** Xihe[25], and **(k)** Dongwan[26] sections. Summer monsoon precipitation increases across the MPB (shaded).

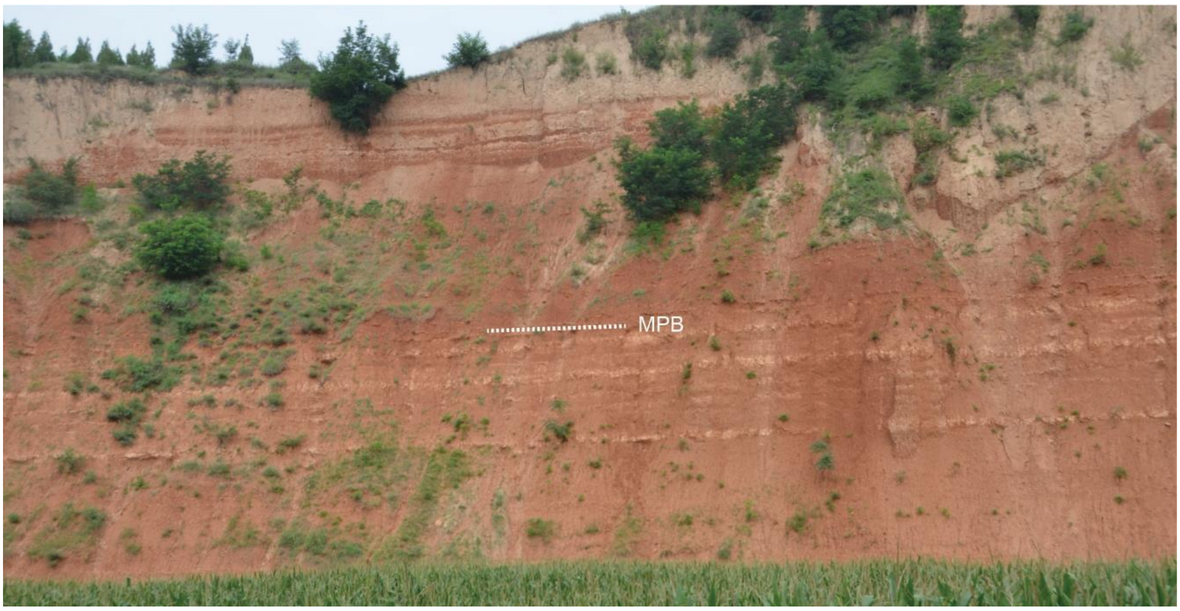

Supplementary Fig. 9. Field photographs of the Linxian red clay on the Chinese Loess Plateau (taken by H. Ao). The upper part (Pliocene) has a more saturated red colouration than the lower part (late Miocene), which is consistent with enhanced pedogenesis and increased summer monsoon precipitation across the Miocene–Pliocene boundary (MPB) at this location.

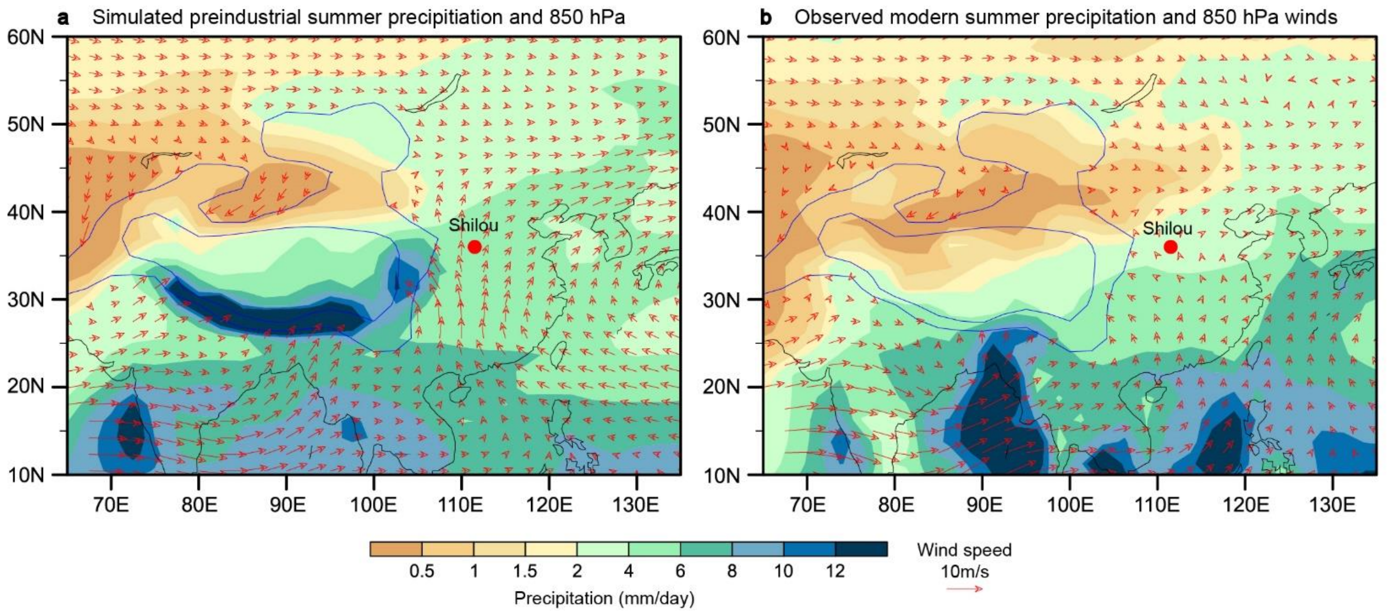

Supplementary Fig. 10. Comparison of simulated and observed summer precipitation. (**a**) Simulated preindustrial summer precipitation (shading) and 850 hPa winds (red vectors) with Community Earth System Model (CESM 1.0.4)[27]. (**b**) Observed modern summer precipitation (shading) from the Climate Prediction Center Merged Analysis of Precipitation (CMAP) reanalysis and 850 hPa winds (red vectors) from the National Center for Environmental Prediction reanalysis II (NCEP II) between 1979 and 2009. Summer consists of May, June, July, August, and September.

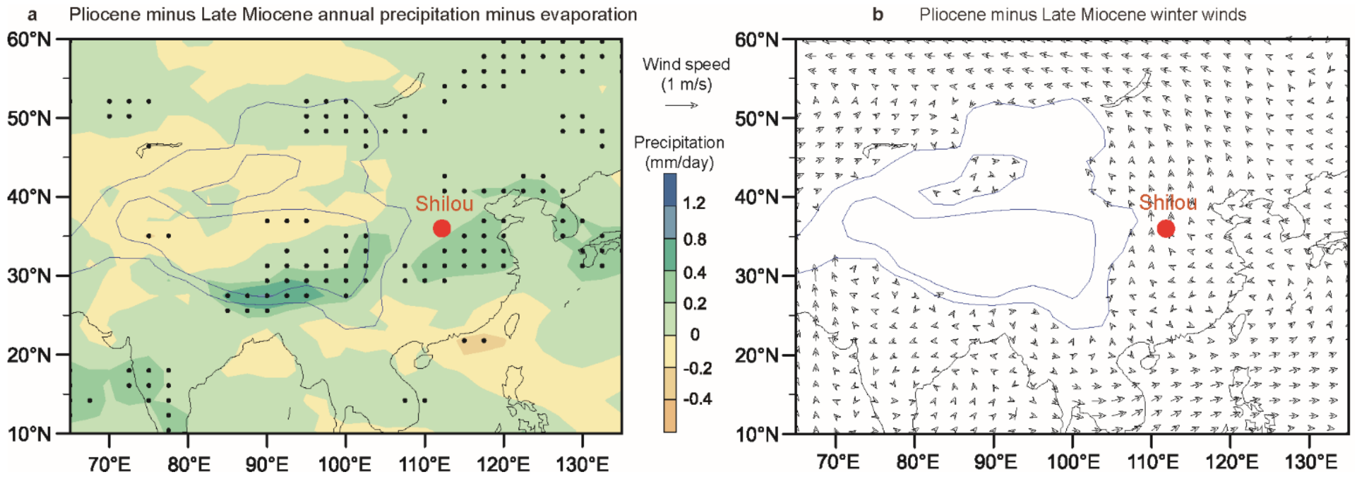

Supplementary Fig. 11. Simulated climate changes in response to CO_2 increase. Simulated changes in response to a CO_2 increase from the late Miocene level of 280 ppm to the Pliocene level of 400 ppm for (**a**) annual precipitation minus evaporation and (**b**) winter 850 hPa winds. Black dots in (a) denote regions statistically significant above the 95% confidence level (Student's *t*-test). Winter consists of November, December, January, February, and March.

Supplementary Table 1. Comparison of estimated ages for palaeomagnetic reversal boundaries in the intermediate (finally selected), youngest, and oldest tuned age models with the 2012 GPTS[18].

Magnetic reversal boundaries	Depth (m)	Depth uncertainty (m)	2012 GPTS (Ma)	Selected age model		Youngest tuned age model		Oldest tuned age model	
				Age (Ma)	Difference (Ma)	Age (Ma)	Difference (Ma)	Age (Ma)	Difference (Ma)
C2An.2r-C2An.3n	3.60	0.02	3.596	3.563	-0.033	3.561	-0.035	3.657	0.061
C2An.3n-C2Ar	8.80	0.02	4.187	4.137	-0.050	4.122	-0.065	4.236	0.049
C2Ar-C3n.1n	9.80	0.02	4.300	4.287	-0.013	4.287	-0.013	4.377	0.077
C3n.1n-C3n.1r	11.00	0.02	4.493	4.487	-0.006	4.421	-0.072	4.505	0.012
C3n.1r-C3n.2n	12.00	0.02	4.631	4.612	-0.019	4.518	-0.113	4.615	-0.016
C3n.2n-C3n.2r	13.80	0.02	4.799	4.807	0.008	4.703	-0.096	4.801	0.002
C3n.2r-C3n.3n	14.20	0.02	4.896	4.822	-0.074	4.734	-0.162	4.821	-0.075
C3n.3n-C3n.3r	17.00	0.02	4.997	5.031	0.034	4.956	-0.041	5.069	0.072
C3n.3r-C3n.4n	19.20	0.02	5.235	5.270	0.035	5.187	-0.048	5.290	0.055
C3n.4n-C3r	28.98	0.01	6.033	6.036	0.003	6.035	0.002	6.052	0.019
C3r-C3An.1n	32.50	0.01	6.252	6.317	0.065	6.199	-0.053	6.318	0.066
C3An.1n-C3An.1r	34.70	0.02	6.436	6.451	0.015	6.381	-0.055	6.461	0.025
C3An.1r-C3An.2n	40.88	0.02	6.733	6.725	-0.008	6.717	-0.016	6.721	-0.012
C3An.2n-C3Ar	47.90	0.06	7.140	7.086	-0.054	7.074	-0.066	7.082	-0.058
C3Ar-C3Bn	50.00	0.04	7.212	7.214	0.002	7.211	-0.001	7.219	0.007
C3Bn-C3Br.1r	50.70	0.02	7.251	7.262	0.011	7.266	0.015	7.277	0.026
C3Br.1r-C3Br.1n	51.15	0.05	7.285	7.287	0.002	7.291	0.006	7.303	0.018
C3Br.1n-C3Br.2r	53.87	0.01	7.454	7.409	-0.045	7.396	-0.058	7.402	-0.052
C3Br.2r-C3Br.2n	54.05	0.01	7.489	7.420	-0.069	7.406	-0.083	7.414	-0.075
C3Br.2n-C3Br.3r	55.50	0.02	7.528	7.510	-0.018	7.490	-0.038	7.507	-0.021
C3Br.3r-C4n.1n	59.03	0.01	7.642	7.591	-0.051	7.565	-0.077	7.587	-0.055
C4n.1n-C4n.1r	59.75	0.03	7.695	7.614	-0.081	7.590	-0.105	7.611	-0.084
C4n.1r-C4n.2n	78.10	0.04	8.108	8.120	0.012	8.120	0.012	8.130	0.022

Supplementary Table 2. Age correlation points used to derive an astronomically tuned age model for the Shilou red clay.

Depth (m)	Age (Ma)	Depth uncertainty (m)	Age,- limit (Ma)	Age,+ limit (Ma)	Negative uncertainty (- Myr)	Positive uncertainty (+ Myr)
1.40	3.420	0.01	3.410	3.520	0.010	0.100
3.00	3.530	0.01	3.530	3.590	0.000	0.060
4.34	3.605	0.01	3.600	3.740	0.005	0.135
5.14	3.660	0.01	3.660	3.880	0.000	0.220
5.68	3.780	0.01	3.770	3.980	0.010	0.200
6.72	3.920	0.01	3.920	4.080	0.000	0.160
8.12	4.080	0.01	4.070	4.160	0.010	0.080
9.18	4.170	0.01	4.150	4.280	0.020	0.110
9.74	4.280	0.01	4.280	4.370	0.000	0.090
10.42	4.390	0.01	4.356	4.441	0.034	0.051
11.32	4.540	0.01	4.460	4.540	0.080	0.000
12.14	4.630	0.01	4.530	4.630	0.100	0.000
12.84	4.750	0.01	4.620	4.760	0.130	0.010
13.72	4.800	0.01	4.700	4.800	0.100	0.000
14.54	4.835	0.01	4.760	4.835	0.075	0.000
15.54	4.850	0.01	4.800	4.880	0.050	0.030
16.42	4.890	0.01	4.870	4.990	0.020	0.100
17.34	5.110	0.01	5.010	5.116	0.100	0.006
18.16	5.230	0.01	5.120	5.230	0.110	0.000
19.20	5.270	0.01	5.190	5.290	0.080	0.020
20.18	5.350	0.01	5.270	5.360	0.080	0.010
21.62	5.460	0.01	5.330	5.460	0.130	0.000
22.96	5.570	0.01	5.450	5.570	0.120	0.000
24.28	5.650	0.01	5.570	5.650	0.080	0.000
25.44	5.750	0.01	5.660	5.750	0.090	0.000
26.52	5.875	0.01	5.770	5.875	0.105	0.000
27.44	5.980	0.01	5.880	5.980	0.100	0.000
28.98	6.035	0.01	6.035	6.050	0.000	0.015
30.42	6.150	0.01	6.120	6.160	0.030	0.010
32.40	6.310	0.01	6.190	6.310	0.120	0.000
34.14	6.440	0.01	6.355	6.450	0.085	0.010
35.94	6.480	0.01	6.435	6.490	0.045	0.010
37.72	6.530	0.01	6.530	6.540	0.000	0.010
39.50	6.650	0.01	6.640	6.660	0.010	0.010
41.50	6.750	0.01	6.750	6.750	0.000	0.000
42.56	6.820	0.01	6.810	6.820	0.010	0.000
44.28	6.910	0.01	6.905	6.910	0.005	0.000
46.78	7.035	0.01	7.030	7.035	0.005	0.000
48.88	7.120	0.01	7.110	7.120	0.010	0.000
50.04	7.220	0.01	7.215	7.220	0.005	0.000
50.92	7.280	0.01	7.280	7.295	0.000	0.015
53.84	7.400	0.01	7.395	7.400	0.005	0.000
55.50	7.510	0.01	7.490	7.510	0.020	0.000
59.04	7.590	0.01	7.565	7.590	0.025	0.000
61.08	7.660	0.01	7.640	7.660	0.020	0.000
66.08	7.740	0.01	7.730	7.860	0.010	0.120
69.02	7.840	0.01	7.820	7.950	0.020	0.110
71.88	7.940	0.01	7.940	8.030	0.000	0.090
75.40	8.046	0.01	8.030	8.070	0.016	0.024
78.10	8.120	0.01	8.120	8.130	0.000	0.010

Supplementary References

1 Ao, H. et al. Late Miocene–Pliocene Asian monsoon intensification linked to Antarctic ice-sheet growth. *Earth Planet. Sci. Lett.* **444**, 75–87 (2016).

2 Guo, Z. T. et al. Onset of Asian desertification by 22 Myr ago inferred from loess deposits in China. *Nature* **416**, 159–163 (2002).

3 Guo, Z. T. et al. A major reorganization of Asian climate by the early Miocene. *Clim. Past* **4**, 153–174 (2008).

4 An, Z. S., Kutzbach, J. E., Prell, W. L. & Porter, S. C. Evolution of Asian monsoons and phased uplift of the Himalaya-Tibetan plateau since Late Miocene times. *Nature* **411**, 62–66 (2001).

5 Ao, H. et al. Mineral magnetic record of the Miocene–Pliocene climate transition on the Chinese Loess Plateau, North China. *Quat. Res.* **89**, 619–628 (2018).

6 Sun, Y. B., He, L., Liang, L. J. & An, Z. S. Changing color of Chinese loess: geochemical constraint and paleoclimatic significance. *J. Asian Earth Sci.* **40**, 1131–1138 (2011).

7 Chen, J., An, Z. S. & Head, J. Variation of Rb/Sr ratios in the loess-paleosol sequences of central China during the last 130,000 years and their implications for monsoon paleoclimatology. *Quat. Res.* **52**, 269–269 (1999).

8 Chen, J. et al. Characterization of the Chinese loess-paleosol stratigraphy by whiteness measurement. *Palaeogeogr. Palaeoclimatol. Palaeoecol.* **183**, 287–297 (2002).

9 Yang, S. L., Ding, F. & Ding, Z. L. Pleistocene chemical weathering history of Asian arid and semi-arid regions recorded in loess deposits of China and Tajikistan. *Geochim. Cosmochim. Acta* **70**, 1695–1709 (2006).

10 He, T. et al. Carbonate leaching processes in the Red Clay Formation, Chinese Loess Plateau: fingerprinting East Asian summer monsoon variability during the late Miocene and Pliocene. *Geophys. Res. Lett.* **40**, 194–198 (2013).

11 Sun, Y. B. et al. Source-to-sink fluctuations of Asian aeolian deposits since the late Oligocene. *Earth-Sci. Rev.* **200**, doi: 10.1016/j.earscirev.2019.102963 (2020).

12 Ao, H. et al. Two-stage mid-Brunhes climate transition and mid-Pleistocene human diversification. *Earth-Sci. Rev.* **210**, doi: 10.1016/j.earscirev.2020.103354 (2020).

13 Xu, Y. et al. An 11-Ma-old red clay sequence on the Eastern Chinese Loess Plateau. *Palaeogeogr. Palaeoclimatol. Palaeoecol.* **284**, 383–391 (2009).

14 Anwar, T., Kravchinsky, V. A. & Zhang, R. Magneto- and cyclostratigraphy in the red clay sequence: new age model and paleoclimatic implication for the eastern Chinese Loess Plateau. *J. Geophys. Res.* **120**, 6758–6770 (2015).

15 Ao, H. et al. Reply to Zhang et al.: Late Miocene–Pliocene magnetochronology of the Shilou Red Clay on the eastern Chinese Loess Plateau. *Earth Planet. Sci. Lett.* **503**, 252–255 (2018).

16 Lisiecki, L. E. & Raymo, M. E. A Pliocene-Pleistocene stack of 57 globally distributed benthic $\delta^{18}O$ records. *Paleoceanography* **20**, PA1003, doi: 10.1029/2004PA001071 (2005).

17 Laskar, J., Fienga, A., Gastineau, M. & Manche, H. La2010: a new orbital solution for the long-term motion of the Earth. *Astron. Astrophys.* **532**, doi: 10.1051/0004-6361/201116836 (2011).

18 Hilgen, F. J., Lourens, L. J. & van Dam, J. A. in *The Geologic Time Scale 2012* (eds Gradstein, F.M., Ogg, J.G., Schmitz, M. & Ogg, G.) 923–978 (Elsevier Press, 2012).

19 Li, M. S., Hinnov, L. & Kump, L. Acycle: time-series analysis software for paleoclimate research and education. *Comput. Geosci.***127**, 12–22 (2019).

20 Sun, J. M. et al. Extreme aridification since the beginning of the Pliocene in the Tarim Basin, western China. *Palaeogeogr. Palaeoclimatol. Palaeoecol.* **485**, 189–200 (2017).

21 Ge, J. Y. et al. Major changes in East Asian climate in the mid-Pliocene: triggered by the uplift of the Tibetan Plateau or global cooling? *J. Asian Earth Sci.* **69**, 48–59 (2013).

22 Zhu, Y. M. et al. A new magnetostratigraphic framework for late Neogene *Hipparion* Red Clay in the eastern Loess Plateau of China. *Palaeogeogr. Palaeoclimatol. Palaeoecol.* **268**, 47–57 (2008).
23 Yang, S. L. et al. A strengthened East Asian Summer Monsoon during Pliocene warmth: evidence from 'red clay' sediment at Pianguan, northern China. *J. Asian Earth Sci.* **155**, 124–133 (2018).
24 Qiang, X. K., Li, Z. X., Powell, C. M. & Zheng, H. B. Magnetostratigraphic record of the Late Miocene onset of the East Asian monsoon, and Pliocene uplift of northern Tibet. *Earth Planet. Sci. Lett.* **187**, 83–93 (2001).
25 Ge, J. Y. et al. Magnetostratigraphy of the Xihe loess-soil sequence and implication for late Neogene deformation of the West Qinling Mountains. *Geophys. J. Int*. **189**, 1399–1408 (2012).
26 Hao, Q. Z. & Guo, Z. T. Magnetostratigraphy of a late Miocene–Pliocene loess-soil sequence in the western Loess Plateau in China. *Geophys. Res. Lett.* **31**, doi: 10.01029/02003GL019392 (2004).
27 Gent, P. R. et al. The Community Climate System Model Version 4. *J. Clim.* **24**, 4973–4991 (2011).
