# Supplementary material for: Global warming-induced Asian hydrological climate transition across the Miocene–Pliocene boundary
Source: Nat Commun. 2021 Nov 26;12:6935. doi: 10.1038/s41467-021-27054-5 (PMC8626456; doi:10.1038/s41467-021-27054-5)
Supplement: Supplementary file 3 — Description of Additional Supplementary Files [file 41467_2021_27054_MOESM3_ESM.pdf]

Description of Additional Supplementary Files

File Name: Supplementary Data 1
Description: Late Miocene-Pliocene summer monsoon proxxy records
